# Starvation in the Midst of Plenty: Reflections on the History and Biology of Insulin and Leptin

**DOI:** 10.1210/er.2018-00179

**Published:** 2018-10-23

**Authors:** Jeffrey S Flier

**Affiliations:** Department of Medicine and Department of Neurobiology, Harvard Medical School, Boston, Massachusetts

## Abstract

Insulin and leptin are critical metabolic hormones that play essential but distinct roles in regulating the physiologic switch between the fed and starved states. The discoveries of insulin and leptin, in 1922 and 1994, respectively, arose out of radically different scientific environments. Despite the dearth of scientific tools available in 1922, insulin’s discovery rapidly launched a life-saving therapy for what we now know to be type I diabetes, and continually enhanced insulin therapeutics are now effectively applied to both major forms of this increasingly prevalent disease. In contrast, although the discovery of leptin provided deep insights into the regulation of central nervous system energy balance circuits, as well as an effective therapy for an extremely rare form of obesity, its therapeutic impact beyond that has been surprisingly limited. Despite an enormous accumulated body of information, many important questions remain unanswered about the mechanisms of action and role in disease of both hormones. Additionally, although many decades apart, both discoveries reveal the complexities inherent to scientific collaboration and the assignment of credit, even when the efforts are spectacularly successful.

**Essential Points**

- Insulin and leptin, discovered 73 years apart, are critical metabolic hormones that play distinct, nonredundant roles in regulating the physiologic switch between the fed and starved states
- The paths to discovery of insulin and leptin were radically different, reflecting the state of biologic science in 1921 vs 1994, but collaboration was required in both cases, and in both cases, effective collaboration was followed by conflict within the teams
- Insulin therapy for diabetes has been continuously enhanced and grown in application since its discovery, whereas leptin therapy is limited today to a very small group of patients lacking leptin and a somewhat larger group with lipodystrophy
- Both insulin and leptin continue to be intensively studied, and although large amounts of new knowledge continue to accrue, key unanswered questions about the mechanisms of action and role in disease of these hormones remain unanswered

The history of endocrinology often begins with accounts of the discovery of hormones. These discoveries enable subsequent research into the physiology, mechanisms of action, and roles of hormones in pathogenesis and therapeutics of disease. Insulin and leptin are two critical hormonal regulators of metabolism. Discovered 73 years apart, in 1921 and 1994, examination of the similarities and differences between these venerable hormones provides important insights into the history of these molecules as well as the broader evolution of metabolic science.

Insulin and leptin play very different roles in human physiology, and, accordingly, children lacking these hormones have starkly disparate phenotypes (Fig. 1) (1, 2). In 1923, an emaciated child with diabetes whose appearance suggested starvation was transformed to normal appearance by daily injections of insulin [Fig. 1(a)] (1). In 1998, a massively obese child had his malady reversed by a course of daily leptin injections [Fig. 1(b)] (2). Although starvation and obesity could not, on the surface, be more different, starvation is actually a key physiologic feature of both insulin and leptin deficiencies. Type I diabetes has been described as “starvation in the midst of plenty.” Why? Despite hyperphagia and high levels of circulating fuels, insulin deficiency prevents effective use of fuels by many tissues, hence “starving” them of nutrition. However, despite their massive obesity, the physiology of leptin-deficient children also entails starvation in the midst of plenty. In this case, primary leptin deficiency causes brain centers that control hunger and energy balance to “believe” the body is starving, and these centers drive unrestrained hyperphagia and weight gain despite increased adipose stores.

**Figure 1.**
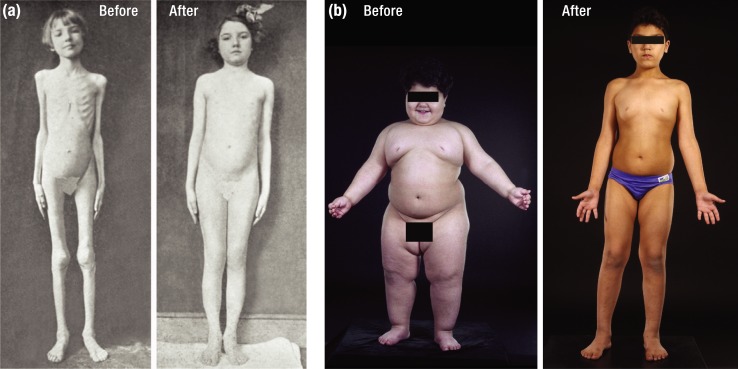
(a) Before and after pictures of a child treated with insulin in 1922. From Bliss (1). (b) Before and after pictures of recombinant leptin treatment in a patient with congenital leptin deficiency. Before, 3 years old weighing 42 kg. After, 7 years old weighing 32 kg. [Reproduced with permission from Farooqi IS, O'Rahilly S. Twenty years of leptin: human disorders of leptin action. J Endocrinol 2014; 223(1):T63–T70. (2)] [© 2019 Illustration Presentation ENDOCRINE SOCIETY]

Despite insulin and leptin playing key roles in adaptation to starvation, these hormones are quite distinct in other respects. The premise of this article is that much can be learned by comparing and contrasting insulin and leptin—the scientific contexts within which their discoveries arose, their paths to discovery at the University of Toronto and Rockefeller University, their development as therapeutics, and selected aspects of their physiology, mechanisms of action, and pathophysiology.

## Scientific Context and Discoveries of Insulin and Leptin

### Diabetes and the discovery of insulin

The discovery of insulin is among the most fascinating stories in the history of science, best recounted in the book *The Discovery of Insulin*, by Canadian historian Michael Bliss (1). Diabetes had been known as a disease since ancient times, when sweet taste of the urine was identified as a cardinal feature. Although its cause was unknown, in the 19th century several lines of evidence suggested a connection of diabetes to dysfunction of the pancreas. Most importantly, in 1889, von Meering and Minkowski reported that removal of the dog pancreas caused blood sugar levels to rise, followed by coma and death, resembling diabetes in humans. A number of scientists around the world then sought to identify a pancreatic extract capable of lowering glucose levels after administration to experimental animals. Despite some promising results, none was definitive, and the hypothesis remained unproven.

In 1920, when Fred Banting initiated his quest to find a pancreatic “internal secretion” in Toronto, medical physiology was very early on the path to becoming a modern scientific discipline, and it was unrecognizable from its state today. There were few physicians and scientists in universities, hospitals, and institutes trained to conduct experiments capable of interrogating physiology and disease. Available tools were rudimentary, as was understanding of biology at the level of molecules, cells, and systems. Physiology was relatively advanced among the scientific disciplines, so in spite of limited tools and understanding, discoveries of lasting significance were made during this era, including the discovery of the first hormone, secretin, in 1902 (3).

Enter Frederick Banting, a scientifically untrained surgeon, recently returned from decorated surgical service in World War I, and seeking, with limited success, to establish a surgical practice. After reading an article on the pancreas and diabetes in a surgical journal, he became preoccupied by the idea that he was capable of extracting the hypothesized factor from dog pancreas. Banting’s motivating idea was that tying off the pancreatic duct of dogs to damage the exocrine pancreas would facilitate production of an effective extract. This idea had been proposed earlier by others, but Banting was unaware of this (1). Ironically, although the idea was logical and motivated Banting’s work, duct ligation was in the end not necessary for success of the project.

By force of will, Banting convinced an appropriately skeptical Professor of Physiology at the University of Toronto, J. J. Macleod, to assist him. Macleod was an expert on metabolism who was familiar with prior reported efforts to discover an antidiabetic pancreatic factor. Although highly skeptical of Banting’s untutored approach and doubtful of his capacity to succeed, he nonetheless provided Banting access to a laboratory, a medical student assistant (Charles Best), some dogs with which to initiate the experimental work, and suggestions (downplayed by Banting) on analytical and extraction techniques. Banting’s approach to preparing tissue extracts was of course primitive by modern standards. Given the abundance of proteolytic enzymes in pancreas, it is remarkable that he and Best obtained positive results, if only erratically, in their diabetic dogs. It was even more remarkable that in January of 1922, an extract they prepared successfully lowered blood glucose in a diabetic child. At a key moment during this period, when the extraction procedure had become irreproducible, Macleod suggested that James Collip, an accomplished biochemist, be added to the team to improve the extraction procedure. Collip’s efforts enabled a reproducible and clinically effective extract that was used by the team in patients with diabetes (1).

What counted as the initial report and publication of the discovery has been a matter of much discussion. Banting and Best coauthored a paper titled “The internal secretion of the pancreas,” presented locally and then published in the *Journal of Laboratory and Clinical Medicine* in 1922 (4). Bliss considered the definitive presentation of the work, the one that convinced the scientific community that the Toronto group had indeed discovered a pancreatic factor capable of treating diabetes, to have been a presentation by Macleod on 3 May 1922, at the Washington, DC meeting of the Association of American Physicians. Many luminaries in the field were in attendance, some of whom had previously tried similar experiments, and Macleod received a standing ovation when he concluded (1). The authors listed on the abstract were Banting, Best, and Macleod, although, unfortunately, Banting and Best did not attend. A paper based on the lecture was published thereafter in the *American Journal of Physiology* (5).

It is well documented that Banting and Macleod disliked each other. Macleod had little regard for Banting’s experimental skill and physiologic understanding. Banting thought that Macleod’s contributions were limited to providing resources. He feared Macleod would use his superior position and reputation to claim excessive credit for the work, and took his Washington, DC presentation as evidence for this. Bliss concluded that Banting’s negative view of Macleod’s personality and motivation was inconsistent with dominant contemporaneous views, and he judged Macleod’s contribution to the discovery to have been important (1).

News of the therapeutic success of the extract in diabetic children, and “the discovery of insulin,” a term chosen from the Latin root insula, for island (of Langerhans), spread rapidly around the world, to both the medical community and the general public. The discovery quickly came to the attention of the Nobel Prize Committee. They carefully examined the work, and in 1923 the Nobel Prize in Physiology or Medicine was awarded to Banting and Macleod. As recounted by Bliss (1), Banting became furious upon hearing this, initially declaring he would not accept a Nobel Prize to be shared with Macleod. He was convinced to accept it by a Canadian government official who suggested that as the first Canadian-born citizen to be awarded a Nobel Prize, he had a duty to Canada to accept it. Banting responded by announcing he would share his half of the award with Best, after which Macleod quickly announced he would share his half with Collip (1). Thus, although recognition for the discovery was complicated by anger and resentment among the participants, public credit for the discovery extended beyond the two scientists selected for recognition by the Nobel Assembly. As described below, credit has evolved with the passage of time.

Commercial and financial interests can influence such credit disputes, but money appears to have played little or no role in the dispute over credit for the discovery of insulin. The patent for insulin was awarded to Banting, Best, and Collip, who quickly sold it to the University of Toronto for 1 dollar (1). By standards of the day, the University received substantial royalties on sales of insulin and used these funds to support both diabetes research and more general university needs (1). No policies existed at the time to guide the sharing of institutional royalties with inventors.

Banting was greatly honored in Canada for his contributions, and he was provided a stipend and funds to pursue research at the University of Toronto (1). The results of his subsequent research were very limited, and he died after a plane crash in 1941 at the age of 49 (1). Macleod continued metabolic research in Toronto for several years, but returned to his native Scotland in 1928, where he served as chair of physiology and eventually dean at Aberdeen Medical School. He made a number of subsequent physiologic discoveries, but did no further work on insulin, and died in 1935 at the age of 58. In 1929, Best succeeded Macleod as Professor of Physiology at the University of Toronto, becoming a major figure in the Canadian scientific community, although his subsequent research had limited impact. Collip returned to his home in Edmonton to pursue endocrine research. He chaired Biochemistry at McGill from 1928 to 1941, and then became dean of Medicine at the University of Western Ontario. He was a pioneer of endocrine biochemistry, doing important research on parathyroid hormone (6). Collip died in 1965 at the age of 72.

After the discovery of insulin, these four scientists had limited interactions, although Banting and Collip eventually became friends. Each judged himself to have been properly considered a discoverer of insulin. Banting’s anger at Macleod never abated. Although Macleod rarely spoke about his dealings with Banting, the dysfunctional nature of these interactions led him to view his Toronto experience in a negative light, despite having enabled his Nobel Prize and attendant fame.

How is scientific credit for the discovery of insulin seen today? Despite the Nobel Prize being awarded to Banting and Macleod, Banting and Best are the dominant names associated today with the discovery of insulin, universally cited in the scientific literature in this regard. Banting unquestionably initiated the Toronto effort and pursued it with dogged commitment. Without him, insulin would not have been discovered in Toronto (1). The Banting Award is the highest award for science bestowed by the American Diabetes Association, and the research institute at the University of Toronto where the work was carried out is named the Banting and Best Institute. When over the years I have informally queried people in medicine and endocrinology about “who discovered insulin,” by far the most common answer is “Banting and Best.” Remarkably, despite having won the Nobel Prize with Banting for this discovery, few today recall MacLeod’s name or associate it with the discovery. Bliss concluded that Macleod’s contributions, although aggressively dismissed by Banting, were significantly undervalued, and the “discovery” would not have been made in Toronto without them. I suspect he would have been treated differently by history had he remained in Toronto. Best is accorded credit for his role in the discovery, as the intrepid colleague working diligently beside Banting in the laboratory, helping to solve problems as they arose (1). Collip contributed essential improvements to the extraction technique that allowed reproducible results without which the project may well have ended unsuccessfully. He was clearly the most accomplished scientist based on his work after the discovery of insulin.

Bliss makes a convincing case that the “discovery” of insulin in Toronto in 1921 required contributions from each of the four individuals above (1). Of course, their work built on the contributions of many others over prior decades, and it benefitted as well from an environment at the University of Toronto that was conducive to the work. Bliss concluded that although their individual contributions were distinct, Banting, Best, McLeod, and Collip should all be considered discoverers of insulin (1). He quoted the view of Lewellys Barker (1) that as regards credit for the discovery of insulin, there was “glory enough for all.”

It is remarkable that total ignorance of the chemical identity of the new factor, which they named insulin, failed to thwart its rapid therapeutic development. Eli Lilly & Company produced insulin from extracts of pig and cow pancreata at an industrial scale, commercializing it within a year of its discovery, followed closely by the Nordisk Insulinlaboratorium in Denmark (1). This speed reflected the pressing needs of dying children, the remarkable skill of the Lilly and Nordisk teams, and a regulatory environment more permissive than that of today. Dramatic “before and after” pictures and widely disseminated human interest stories promoted the idea that this discovery should be exploited rapidly in the service of human health.

It is also worth considering what the discovery of insulin did not reveal about this molecule. Although “discovered” in 1921, insulin’s identity as a two-chain polypeptide hormone of defined amino acid sequence was not established until 28 years later (Table 1) (7). This required the pioneering research of Fred Sanger, who invented new methods to accomplish the task. He chose insulin because, as the first therapeutic protein, he could purchase it. Sanger received the first of his two Nobel Prizes for this work in 1949 (7). Insulin levels in blood were not measurable until 1959, 38 years after its discovery (Table 1). Yalow and Berson (8) described the radioimmunoassay method, first applied to insulin and then more broadly. For this, Yalow received the Nobel Prize in 1977, Berson having died in 1972. Their discovery arose from the prior demonstration that insulin-treated diabetic patients had anti-insulin antibodies, and their insight that such antibodies might be employed to detect and quantitate the hormone. The cellular mechanism of insulin action also took many years to be clarified, with years of uncertainty as to whether the hormone’s initial molecular target was on the cell surface or inside cells, where it was known to modify the activity of several enzymes. High-affinity insulin receptor binding activity was demonstrated on the cell surface in 1972 (9), and in 1975 blockade of receptor binding by autoantibodies to the insulin receptor was shown to cause extreme insulin resistance in rare human patients, further establishing the essential role of these receptors (10). Using these same autoantibodies, the tyrosine kinase activity of the receptor was identified several years before the protein was purified or the gene was cloned (11, 12), an order of discovery very different from the typical contemporary paradigm.

**Table 1.** Comparative History and Biology of Insulin and Leptin

	Insulin	Leptin
Discovery	1922 University of Toronto	1994 New York/Rockefeller University
	Banting^*a*^/Best/Macleod^*a*^/Collip	Friedman/Leibel/Coleman
Path to discovery	Search for an unknown but hypothesized pancreatic hormone regulating glucose	Search for mutant gene causing severe obesity in mice
Time from discovery to molecular identification	27 y: 1949 (Sanger^*a*^)	Instantaneous
Time from discovery to first clinical use	1 y	5 y
Time from discovery to first assay measurement in blood	38 y (Berson and Yalow^*a*^)	<1 y
Physiologic roles	Metabolic regulation: fed/fasted transition	Fed/fasted transition: hunger; neuroendocrine
	Glucose, lipid, and protein homeostasis	Possible resistance to obesity from overfeeding
Key target organs	Liver, muscle, fat (plus many others of less clear importance)	Hypothalamic neurons, immune cells
Clinical indications for therapy	Diabetes (type 1 and type 2)	Genetic leptin deficiency
		Lipodystrophies
Pathophysiologic links to disease	Type 1: autoimmune—*β* cell destruction	Rare patients with obesity due to mutant leptin gene, or mutant leptin receptor gene
	Type 2: resistance to insulin action plus insufficient insulin secretion	Rare lipodystrophies (lack of fat tissue) → low leptin levels
	Rare mutations of insulin or its receptor, or blocking antibodies to insulin receptor	Common obesity with resistance to leptin action
		Possible subset of obesity with relative leptin deficiency
Patients under treatment	Tens of millions with diabetes	Tens to thousands with genetic obesity or lipodystrophy
Developmental milestones as therapeutic Agent	Animal organ extract → adjuvants (protamine) → recombinant human → novel analogs with unique pharmacokinetics	Initial recombinant human analog

^a^ Awarded the Nobel Prize.

Today, research on insulin action and physiology continues, with 377,000 papers in the PubMed database since 1921, and 17,000 in 2017. Although many questions about insulin have been answered, many important questions remain to be solved.

### Obesity and the discovery of leptin

Obesity has been known as a medical condition since antiquity (13). During long periods when humans struggled with food scarcity, obesity was uncommon, and when it did exist it was associated with wealth, and with fertility in women. In more recent times, and especially recent decades, the prevalence of obesity has increased dramatically, and its adverse effects on human health are now widely recognized (14, 15). Research to clarify the etiology and physiology of obesity lagged behind many other disorders, including diabetes, in part because physiological and biochemical tools to study the causes of obesity were limited, especially in humans. Apart from typically ineffective dietary advice, safe and effective treatments were largely nonexistent, and for a major part of the 20th century, the study of obesity contributed relatively little to leading-edge biomedical research. This began to change as family and twin studies revealed that—alongside availability of food and aspects of the environment, including the demand for physical labor—inheritance played an important role in this disorder (16). The role of genes was also supported by the identification of rodent strains with autosomal recessive syndromes of extreme obesity (17, 18). These mice, most notably *ob* and *db*, were extensively characterized as models for obesity of genetic etiology, but research on these models was slow to produce insights into physiologic regulation of appetite and weight, or a deeper understanding of human obesity.

This changed dramatically with the cloning of the *ob* gene in 1994, a discovery that arose in a radically different scientific context from that prevailing seven decades earlier when insulin was discovered (19). Among these changes, the size of the bioscience research community had dramatically increased by the 1990s, and the scientific workforce had become more professionalized. The tools for investigating biomolecules had dramatically advanced, along with enormously increased understanding of cellular physiology and regulation, including mechanisms of hormone action. DNA was established as the key molecule of inheritance, its structure and the genetic code were elucidated, and cellular mechanisms for synthesizing and regulating proteins were defined (20). As biochemical insights emerged into how these processes orchestrate cellular function and disease, molecular biology was developing as an ascendant discipline. The capacity to identify and express the products of specific genes enabled their function to be studied in previously unimaginable ways.

The stage for the cloning of the *ob* gene was set by Douglas Coleman. His seminal work at Jackson Laboratory established that *ob/ob* and *db/db* mice, identified earlier at Jackson (17), were autosomal recessive disorders characterized by hyperphagia and massive obesity (21). Coleman’s breakthrough involved the use of parabiosis experiments, in which two animals are surgically linked, permitting exchange of circulating molecules. Parabiosis provided compelling physiological evidence that obesity in *ob/ob* mice might be due to lack of a circulating satiety factor, whereas obesity in *db/db* mice might result from an inability to respond to this factor (22). Not surprisingly, given the low concentration of hormones in the blood, efforts to identify such a factor using biochemical approaches were unsuccessful. Given the complexity and potential pitfalls of the parabiosis model, many in the field remained unconvinced by the Coleman hypothesis. Jackson Laboratory made the mice available to interested investigators, most using them to characterize their physiology. More than 1000 papers were published on these mice prior to the gene being cloned.

By 1986, when research to identify the *ob* gene was initiated at Rockefeller University, genetic insights and techniques had just begun to successfully identify disease genes. Initial studies employed candidate gene approaches, in which plausible genes based on known pathways were sequenced. Unbiased genetic approaches, seeking to identify genes purely through genetic linkage, were beginning to be explored in both mice and humans using a variety of mapping techniques (23, 24). Notable successes were identification of the genes for Duchennes muscular dystrophy (25) and cystic fibrosis (26). These efforts required teams of scientists, some trained in emerging genetic methodologies, and such people were then limited in number. Scientists who chose to pursue discovery of disease genes using unbiased approaches could not then be certain about their eventual success. Initiation of such projects required passion for the subject, technical skill, resources, long-term commitment, and a degree of good fortune.

Rudy Leibel and Jeff Friedman were faculty at Rockefeller. Although differing by 12 years in age (Leibel was older) and coming to the problem via different paths, they decided in 1986 to work together on this challenge. Friedman was a talented MD who had recently completed PhD research in the laboratory of James Darnell at Rockefeller, where he worked on hepatic cancer, developing skills in recombinant DNA technology (27, 28). In 1986, he was chosen by Rockefeller leadership to transition to an independent faculty position, an uncommon opportunity at the time, and he selected the genetics of murine obesity as a disease-related topic on which to build his independent research career (29). Leibel was a pediatric endocrinologist whose interest in obesity arose through clinical interactions with obese children. In 1978, 8 years before this collaboration began, he left a faculty position at Harvard Medical School for Rockefeller University to join Jules Hirsch, a prominent obesity researcher (29). Leibel’s research at Rockefeller focused on human adipocyte biochemistry (30–32), and he eventually became an associate professor and co-laboratory head with Hirsch.

Whether Friedman or Leibel first had the idea to clone the *ob* gene, and who first proposed to pursue it collaboratively is uncertain, and may not be important, as the idea itself was not unique. Nevertheless, these two scientists initiated a multiyear collaboration aimed at accomplishing this goal. With complementary skills in molecular genetics and physiologic biochemistry, they appeared well positioned for success (29). The work would combine extensive mouse crosses and metabolic phenotyping with the use of chromosome markers to identify genetic linkages to the *ob* locus, eventually closing in on the gene itself. With technology and approaches then available, this would be an arduous task requiring years to accomplish. Further highlighting the risks that were taken, it is likely that little attention or credit would be accorded the interim steps along the way to the ultimate goal of gene identification, and there was absolutely no assurance of that ultimate success during the effective career of a scientist at the time.

Obtaining funding for such a long-term and uncertain project might have been daunting, but in the Rockefeller environment both Friedman and Leibel were successful, receiving funding from various sources to support the work. Friedman was supported for the *ob* gene cloning by the Howard Hughes Medical Institute (HHMI). Given his junior status when first funded and the years taken for the project goals to succeed, the sustained commitment to Friedman by HHMI was critical to his success and that of the project. Friedman and Leibel were also jointly funded to clone *ob* by a National Institutes of Health RO1 grant, titled “The genomic basis of heritable obesity,” with Friedman as principal investigator and Leibel as co–principal investigator. Friedman and Leibel both sought and obtained additional funding for their work on this topic and related areas. Early on, Leibel obtained a fellowship grant enabling medical student Nathan Bahary to join him and Friedman on the *ob* gene identification project. Bahary pursued his MD/PhD with Leibel and Friedman as co-mentors, published four first-authored papers with them (33–36), and received his PhD for this work from Rockefeller in 1992. High-profile public presentations by both Leibel and Friedman during several years brought progress on their effort to clone *ob* to the attention of the metabolic research community.

The definitive *ob* cloning paper was published in *Nature* in December of 1994, to major and well-deserved international acclaim (19). The subsequent paper naming the gene product leptin (from the Greek root leptos, for thin), and demonstrating that recombinant leptin injections reversed the obesity syndrome in *ob/ob* mice, was published in *Science* in 1995 (37). Friedman was the senior and corresponding author on both papers. Although many in the field were surprised by Leibel’s absence as an author on the 1994 gene identification paper (Leibel and Bahary were thanked in the acknowledgments for assistance in the early stages of the work) (19), Leibel never publicly challenged this outcome, subsequent to which his identification with the *ob* cloning project waned.

In 2002, science journalist Ellen Ruppel Shell published the book titled *The Hungry Gene* after substantial research, including interviews with many of the principals. In chapter 5, she addressed the efforts at Rockefeller University to clone *ob*, and the evolving interactions of Leibel, Friedman, Bahary, and others. She described important changes to the collaboration that took place in 1992/1993 (29). This book is the most detailed and critical published account of what took place at that time, although aspects were discussed in two other books (38, 39).

In Shell’s account, several years into the collaboration and well before the final cloning of *ob*, major problems arose between Friedman and Leibel. Among these, Friedman became increasingly concerned that Leibel might claim and/or be accorded disproportionate credit for the final gene identification, despite what Friedman saw as his own dominant role, especially in the final phase of the work (29). Although important details of this conflict and its origins are unknown to the public apart from Shell’s account, such disputes over credit are hardly unprecedented, and they may be more common when one of two collaborating scientists is better known in the field, as was Leibel in this case. The tendency of better known scientists to receive disproportionate credit for a similar contribution (*e.g.*, “the rich get richer”) was reviewed by the sociologist of science Robert Merton, a phenomenon he named the Matthew Effect (40). Whether the rising tensions between them arose from concerns about credit or other factors, in early 1993 Friedman and Leibel formally agreed to administratively separate their laboratory efforts going forward, including a plan for each to end their prior roles as co-investigators on the others RO1 grants. They agreed that henceforth each would focus on his independent research projects, and Friedman would finalize molecular cloning of *ob* (29). Leibel stepped back from “the day to day involvement. But he remained in close contact with Friedman and his staff, who continued to seek his counsel and advice” (29). Friedman made clear his view that authorship on the *ob* gene identification paper would be based on his assessment of specific contributions to the work to be reported in that paper. Leibel always assumed that despite this agreement, both he and Bahary would be co-authors on the final cloning paper (29).

*“How has credit for the discovery of leptin been acknowledged during the past 24 years?”*

Assignment of proper credit for major discoveries, such as insulin and leptin, is important to individual scientists involved in the work and to the broader scientific community. Nevertheless, uncertainties commonly arise in assigning authorship and credit for scientific discoveries. These may reflect many factors, including scientific or personal conflicts among participants, contentious aspects of institutional culture, behaviors linked to credit or potential for financial reward, disputed facts regarding who did what at what time and the value of individual contributions, and ambiguities regarding standards for authorship and awarding credit. Apportioning credit is especially complex when involving interdisciplinary teams, rather than scientists working in isolation, and in projects of long duration where intermediate steps may be largely forgotten or discounted while the “credit-worthy” discovery is recorded in a final publication.

How has credit for the discovery of leptin been acknowledged during the past 24 years? The 1994 *Nature* paper is universally regarded as “the *ob* gene discovery paper.” Because Friedman is the sole faculty author and this outcome has not been explicitly contested, it is not at all surprising that Friedman has been accorded the credit for cloning of the *ob* gene. Several papers recording progress toward that goal, which had Bahary as first and Leibel as a middle author, are part of the historical record, but these are now infrequently referenced in accounts of the discovery of leptin (33, 35, 36). Friedman alone directed several key follow-up studies, including the demonstration that replacing the *ob* gene product leptin in *ob/ob* mice lacking it corrected their obesity (37). This result constituted the definitive proof that the Coleman hypothesis was correct, and that the *ob* gene in fact encoded an adipocyte hormone necessary for regulation of appetite and body weight.

Awards and patents may serve as additional inputs to assigning credit for discoveries. Friedman and Coleman (the latter of whom died in 2014) have been co-recipients of many prestigious awards specifically for their role in leptin’s discovery, including the Shaw Prize, the Gairdner Award, and the Lasker Award, the latter in 2010 (41). By including Coleman together with Friedman, these award committees chose to frame “the discovery” as representing more than the molecular cloning of the gene. These award committees, each with their own due diligence and selection panels of scientific luminaries, did not judge Leibel’s role as meriting similar recognition. Additionally, in 1994, neither Leibel nor Bahary were identified by Rockefeller officials and their patent attorneys as inventors on the patent (42). The initial business agreement with Amgen transferred development rights for leptin in exchange for substantial payments to the institution (Rockefeller and HHMI), shared with the inventors (43). Had leptin become a successful therapy for obesity, royalty payments would have been enormous.

Both Friedman and Leibel have had highly successful careers and continued to conduct important research on obesity and leptin biology, albeit with different emphases and directions. Friedman became a laboratory head at Rockefeller and has long held an HHMI position. Leibel left for Columbia University Medical School, where he is a professor, heads a division of molecular genetics in the department of pediatrics, co-directs the Berrie Diabetes Center, and is the director of the New York Obesity Research Center.

Friedman and Coleman both unquestionably deserve the enormous credit they have received for this discovery, with Friedman recognized as the lead scientist on the molecular cloning of *ob*. That Friedman took on this ambitious long-term project to establish his career was both unusual and deserving of high praise. However, without diminishing the credit accorded to Friedman, some have wondered whether Leibel received the full measure of credit that he deserved for his substantial contribution. Questions about relative assignments of credit are notoriously difficult to resolve. This may require objective historical research to uncover agreements and understandings the two scientists might have had, and critical assessment of Leibel’s role in design and execution of the multiyear strategy for mouse breeding and phenotyping, as well as co-mentorship of key junior scientists. He worked in partnership with Friedman for a period of 4 to 6 years, while Friedman led the critical molecular genetic interrogation of DNA. Perhaps in the future, a historian of science will dive deeply into the discovery of leptin, as Bliss did for insulin, seeking to provide greater detail and context on the people and events that produced this important discovery. At this point, the discoveries of leptin and insulin share the fact that collaborators had a falling-out that, whatever its origins, raised questions about credit that today are not easily resolved.

The pace of scientific events following the discovery of leptin was, not surprisingly, strikingly different from that for insulin. Because the “discovery” of leptin was the identification of the gene whose mutation produced the *ob/ob* mouse, the identification of the encoded protein leptin was a direct consequence of the cloning and sequencing of the gene (Table 1). This contrasts with insulin, whose discovery was the reproducible extraction from pancreas of a chemically unidentified activity that lowered blood glucose, which preceded molecular identification of the protein by 28 years. Similarly, measurement of leptin levels with antibodies to the predicted protein was possible within months of discovery, whereas this took 38 years for insulin (Table 1). In both cases, these therapies reached humans remarkably quickly: less than 1 year for insulin from discovery to first therapy in humans, and only 5 years for leptin from cloning to human therapy (44) (Table 1). Although less rapid than insulin, leptins first clinical use was rapid by contemporary standards for the speed of pharmaceutical development. As with insulin, many questions about leptin have been answered, but 24 years after its discovery, many more remain. From the first paper using the term leptin in 1995 through 2017, 33,000 papers have been published on leptin, with 2200 in 2017 alone.

## Therapeutic Impact of Insulin and Leptin

### Insulin

From its initial discovery, the therapeutic potential of insulin was never in doubt. Insulin was life-saving when administered to patients with what we now know to be type I diabetes. Although some viewed insulin as a cure for diabetes, this was surely not the case. Although saved from death, the life of insulin-treated people with diabetes was not normalized, either on a daily basis, with unpredictable bouts of hypoglycemia and acidosis, or in the long term, as chronic complications of diabetes could now become manifest (45). However, the disease’s most immediate and dramatic implications were forever altered. Over time, it became apparent that type 2 diabetes, the form usually associated with obesity, was far more prevalent. Although not necessary for immediate survival in these patients, insulin therapy reduces both blood glucose and long-term microvasular complications (46). As the prevalence of obesity has risen during recent decades, so has the prevalence of type 2 diabetes, and with it the use of insulin. The etiologic role of *β* cell autoimmunity in type 1 diabetes became evident by the 1980s (47), but a prevention or treatment based on this knowledge has yet to emerge. The incidence of type 1 diabetes also continues to increase (48), and insulin remains an essential therapy in nearly all cases.

The effectiveness of insulin therapy has been enhanced by both development of novel formulations and enhanced capacity to monitor blood glucose levels. Insulin formulations such as neutral protamine Hagedorn permitted more sustained absorption and biological actions, highly purified beef and pork insulins reduced inflammatory and allergic responses, and human insulin became the first recombinant protein therapeutic (49), a major milestone in human therapeutics. In recent decades, engineering the insulin sequence has produced analogs with favorable pharmacokinetics (50). More facile systems for self-administered injection, and insulin pumps, have also been developed (51). Approaches to administering insulin without injection through transnasal, transdermal, or oral routes have been investigated, but these have not been effective or widely adopted. A limited number of patients with type 1 diabetes are successfully treated by islet cell transplantation (52). The global insulin market was estimated at $27 billion in 2015, and it is expected to grow at an annual rate of 8%. In the United States, addition to the >1 million people with type 1 diabetes, ∼30% of the >20 million people diagnosed with type 2 diabetes take insulin, half of these together with an oral agent or other therapy (53).

### Leptin

Although the initial and quite reasonable hope was that leptin would become as dramatic a treatment of obesity as insulin was for diabetes, this has not proven to be the case. It took several years for this to become evident, because the few patients with obesity due to loss-of-function mutations in the leptin gene did indeed have dramatic responses to daily injections of recombinant leptin (44). These patients had rapid reduction in hunger and food intake followed by sustained loss of excess adipose mass, raising hopes that leptin therapy might also be effective in humans with typical obesity. If so, leptin would have become one of the most important therapeutics of all time. However, patients with genetically determined complete leptin deficiency are exceedingly rare, and the latter dream has not been realized.

Early studies in mice (54) and humans (55) revealed that rather than deficiency or absence of the hormone, obesity was typically associated with increased circulating concentrations of leptin, generally proportional to the extent of obesity, although leptin levels do vary over a substantial range at any given body mass index (55, 56). This initially appeared similar to the situation in type 2 diabetes, where obesity, hyperinsulinemia, and insulin resistance coexisted (57). Because in patients with type 2 diabetes insulin therapy retained the ability to lower blood glucose despite hyperinsulinemia and insulin resistance, many hoped that, despite hyperleptinemia, leptin therapy might be effective in typical obesity and obesity-associated diabetes. The first indication to the contrary was when leptin was tested in a commonly studied mouse model of diet-associated obesity, and significant antiobesity effects of peripherally injected leptin were not observed (58). Most importantly, the one large study of leptin therapy for human obesity, conducted by Amgen, failed to achieve its therapeutic endpoint for efficacy in an unselected population with obesity (59). As a result, Amgen abandoned leptin, transferring patent rights to Amylin, which subsequently passed the rights to other companies. Aegerion is the company that today holds rights to the leptin analog metreleptin. At this time, the worldwide impact of leptin on the treatment of human obesity is limited to ∼25 individuals who receive it for congenital leptin deficiency, in whom it continues to work extremely well.

*“In summary, the therapeutic trajectories of insulin and leptin could not, so far, have been more different.”*

Is it possible that a significant subset of patients with obesity and relatively low leptin levels have a state of partial “leptin deficiency” and be responsive to leptin administration? In a longitudinal study of Pima Indians with obesity, some of whom gained weight during a 2-year period and others who did not, those who gained weight had relatively lower leptin levels as a function of fat mass, consistent with the possibility that raising their leptin levels might have prevented weight gain (60). Recently, data from Amgen’s trials conducted 20 years ago have been reanalyzed with that idea in mind. In this reanalysis, those obese individuals whose leptin levels were in the lowest 10th percentile had statistically significant leptin-induced weight loss compared with placebo during 26 weeks; the extent of weight loss was most clinically relevant in those whose leptin levels were in the bottom first percentile (61). This raises the exciting possibility that, using low leptin levels as a biomarker, a leptin analog or other receptor agonist might be developed as a therapeutic for a subset of patients with obesity. Given the high prevalence of obesity, efficacy of leptin in even a small subset of obese patients could be an important therapeutic advance.

Interestingly, another group of patients who benefit from leptin therapy are those with deficient fat mass rather than an excess. The lipodystrophies are a heterogeneous group of relatively uncommon disorders in which low leptin levels result from deficient mass of adipose tissue, the normal source of circulating leptin (62). In these patients, the combination of deficient adipose mass and low leptin level promotes metabolic dysfunction, including hypertriglyceridemia, severe fatty liver, insulin resistance, and, in most cases, diabetic glucose tolerance curves. Leptin therapy produces major metabolic benefits (62), and it is now approved by the US Food and Drug Administration for this indication. A recent paper demonstrated that, in lipodystrophy, the metabolic benefits of leptin occurred independent of suppression of food intake (63). In contrast, administration of leptin for 2 weeks to patients with obesity and newly diagnosed type 2 diabetes had no effect on either fat mass or any measure of glucose metabolism or insulin sensitivity (64). In mouse models of type 1 diabetes, leptin is surprisingly able to reverse hyperglycemia independent of insulin therapy (65, 66). Possible mechanisms in the mouse include glucagon suppression, actions on leptin-responsive hypothalamic neurons, and suppression of the hypothalamic–pituitary adrenal axis (65–67). In a recent study of human patients with type 1 diabetes who had suboptimal glycemic control, metreleptin failed to show an effect to reduce blood glucose during 12 to 20 weeks (68). A modest effect to reduce weight and insulin dose was observed (68).

Future research may eventually identify novel approaches to broaden the utility of leptin therapy in obesity. This could involve: identifying subgroups of obese patients with reduced leptin levels as described above; novel approaches to countering resistance to leptin; or using leptin, not to induce weight loss, but to maintain it after successful dieting. Each of these approaches is plausible, although none has yet been successfully developed. It has been shown that restoring lowered leptin to normal in humans after stable weight loss has several physiologic effects expected to promote resistance to weight regain (69). Following massive weight loss achieved by bariatric surgery or caloric restriction plus vigorous exercise, leptin levels fall markedly, and the percentage change in circulating leptin correlated positively with the suppression of energy expenditure (70).

In summary, the therapeutic trajectories of insulin and leptin could not, so far, have been more different. Insulin was successful from the outset in a large group of patients, and its use and effectiveness have continuously grown. In contrast, leptin’s therapeutic use has, to date, been limited to those with two disorders, one extremely rare and the other quite uncommon.

## Physiology and Pathophysiology of Insulin and Leptin

### Insulin

In the 97 years since its discovery, insulin has been extensively studied, and its physiologic role is well defined (71). Insulin levels rise and fall rapidly in response to feeding and starvation, and in broadest terms, changing insulin levels orchestrate the metabolic switch between anabolism and catabolism. The regulation of insulin secretion and circulating levels is highly complex, reflecting *β* cell mass and secretion. Among the regulators of insulin secretion, glucose is the most important, but other nutrients (free fatty acids, amino acids), hormones (GLP-1, GIP), and sympathetic and cholinergic autonomic influences play important roles in acute regulation of hormone levels (72). The rapid rise of insulin with feeding promotes uptake and storage of ingested energy in fat and muscle, and it suppresses hepatic glucose production, together keeping postprandial blood glucose levels from rising excessively. The rapid fall of insulin with starvation promotes catabolism. Energy stored in fat is released, and glucose production and release from the liver increase, together preventing blood glucose from falling to dangerous levels, thereby protecting the brain, which requires a continuous supply. Insulin has actions on many other tissues and processes, the discussion of which exceeds the scope of this review.

The molecular basis for insulin action has garnered enormous attention from thousands of investigators. Much has been learned about insulin receptors, insulin receptor substrates, and the downstream signaling and effector pathways that together account for the totality of insulin action (73). Because resistance to insulin action on various organs and pathways characterizes type 2 diabetes, and together with inadequate compensatory insulin secretion drives its pathogenesis, many studies have sought mechanistic explanations for insulin resistance. Some components of insulin resistance appear to be inherited (74), and some develop later in life. The genes responsible for the heritable component of insulin resistance in typical type 2 diabetes are, perhaps surprisingly, still largely unidentified (75). Functionally significant variation in genes encoding the canonical signaling components (*e.g.*, insulin receptors, insulin receptor substrates, Akt) have been identified in severe inherited insulin resistance, and, although extremely rare, they have been quite informative (76, 77). Many defects in target cells of diabetic patients have been identified, including reduced receptor density, reduced expression and translocation of glucose transporters, and alterations in diverse signaling pathways (78). Surprisingly, it is still uncertain which of these are most responsible for the insulin resistance in type 2 diabetes, and exactly how and when in the course of disease these pathways become altered. A number of new therapies have been developed for diabetes, but despite the optimistic narratives of thousands of grant proposals, none has arisen through targeted pharmacologic efforts to counter specific molecular mediators of insulin resistance. Metformin, which does lower hepatic glucose output and thereby improves diabetic control, does so without directly improving insulin signaling (79).

### Leptin

In the 24 years since its discovery, the physiology and mechanisms of leptin action have also been the subject of substantial investigation. Both leptin and insulin play key roles in signaling the physiologic transition from energy sufficiency to deficient energy/starvation, but the processes regulated by these hormones and the time courses on which they operate differ. Unlike insulin, which rises rapidly after meals, leptin levels rise gradually in proportion to increasing fat mass. The factors responsible for this regulation are largely at the level of adipocyte leptin mRNA expression. Among the factors shown to regulate leptin expression are glucocorticoids, insulin, and *β* adrenergic receptors (80–82). The fall in leptin levels with fasting occurs over a period of hours and signals starvation to the brain, where leptin-sensitive neurons in the hypothalamus and hindbrain initiate homeostatic responses, including hunger and reduced energy expenditure. A role for proopiomelanocortin neurons and the sympathetic nervous system via the inhibitory adrenergic receptor ADRA2A has recently been described in the fasting-induced fall of leptin (83). Falling leptin suppresses several energy-consuming physiological processes regulated by the neuroendocrine system, including reproduction, thyroid function, and sympathetic autonomic activity (84). These consequences of low leptin are predicted to extend survival by preserving energy stores when energy is scarce (85). When energy intake is restored, leptin levels rise, signaling reversal of these homeostatic adaptations. The physiologic consequences of low leptin levels have been demonstrated in both rodents and humans (84, 86, 87). Although much has been learned about the neural and effector pathways downstream of leptin receptors that mediate these changes (88), much remains to be discovered. Reflecting the physiologic parallels between the two hormones, some actions of insulin on the brain related to energy balance overlap with those of leptin and may be mediated by similar pathways (89).

**Figure 2.**
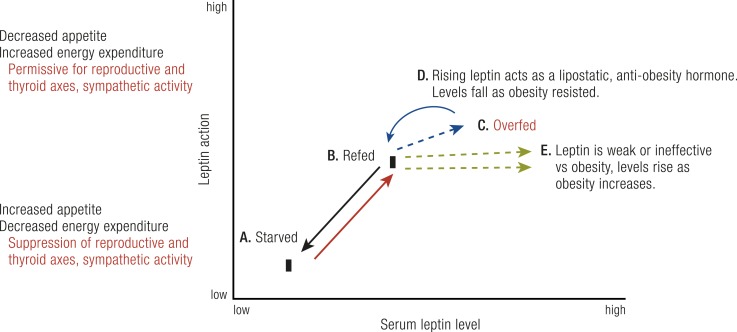
Relationships between serum leptin levels and leptin action. The major effects of changing leptin levels on leptin physiologic actions occur between (A) the low levels seen with food restriction or starvation and (B) the rising levels seen with refeeding. Low levels cause increased hunger, decreased energy expenditure, and suppression of the reproductive and thyroid axes and sympathetic nervous system activity. As levels rise with refeeding, hunger diminishes, sympathetic nervous system activity rises, and reproductive and thyroid axes return to normal. (C) With overfeeding, there are two possible patterns for leptin responsiveness. (D) In one, rising leptin serves as a lipostatic signal, acting on hypothalamic centers to further suppress hunger and/or increase sympathetic activity, reducing weight, limiting obesity, and lowering leptin levels. (E) In the other pattern, which is more common, leptin levels rise further, reflecting fat mass as obesity progresses, but these higher leptin levels exert little or no additional effect to suppress hunger or enhance sympathetic activity to resist obesity. [©2019 Illustration Presentation ENDOCRINE SOCIETY]

That changing leptin levels signal the switch between starvation and energy sufficiency is now well established. Upon its discovery, it was extremely reasonable to expect that further increases in leptin levels occurring with “excess adiposity” might further suppress appetite and increase energy expenditure, thereby serving as a physiologic “lipostatic” signal to resist obesity (90). Consistent with the initial element of this hypothesis, leptin levels do rise as adiposity increases. Because hyperleptinemic obese individuals are much less obese than those completely lacking leptin or its receptor, it is evident that elevated leptin levels in typical obesity do continue to mediate leptin action on energy balance pathways. However, do the higher leptin levels in obesity suppress appetite and increase energy expenditure to a greater extent than do merely “sufficient” leptin levels? What is clear is that endogenous hyperleptinemia in obesity does not suppress appetite and/or increase energy expenditure sufficient to restore these individuals to “normal weight.” This is not only true for endogenous hyperleptinemia; further increasing leptin levels with recombinant leptin did not reduce adiposity significantly in most obese individuals (59). As stated above, a minority of obese individuals who produce less leptin as their fat mass expands may have clinically useful responses to leptin supplementation (61). Further research to identify the molecular basis for relatively low leptin levels and to demonstrate clinical leptin responsiveness of this subgroup and others will be awaited with interest. Overall, in most individuals with obesity, leptin’s action to suppress appetite and increase energy expenditure seems to “saturate” well before the high levels observed in their blood (Fig. 2).

In this regard, the shape of *in vivo* dose-response curves for leptin and insulin appear to diverge, especially at elevated hormone levels. Let us first examine the dose-response curve for insulin action on glucose metabolism. Insulin action to enhance glucose uptake into muscle and fat increases as insulin rises within the physiologic range, as occurs after meals (71). However, when levels are further increased with exogenous insulin, these pathways are further stimulated, which may readily cause hypoglycemia (91). The ability of “supraphysiologic hormone levels” to produce “excess action” beyond homeostatic needs characterizes most hormone systems, accounting for diseases and states of excess hormone action, whether arising from dysregulated endogenous overproduction or exogenous administration.

*“That changing leptin levels signal the switch between starvation and energy sufficiency is now well established.”*

In the case of leptin, the biologic dose-response curve for suppressing hunger and increasing energy expenditure exists within a fairly narrow range, transitioning between a state of energy insufficiency with low leptin levels and energy sufficiency evoked by modestly higher levels (Fig. 2). That further increases in leptin can cause supraphysiologic actions (*e.g.*, marked suppression of appetite and/or further enhanced energy expenditure, together sufficient to cause pathologically excessive weight loss) has been suggested by studies of normal mice infused with leptin (92), but this has yet to be clearly demonstrated via physiologic experiments in humans (93). This narrow dose-response pattern, at least in most humans, has major implications for leptin physiology and obesity. It would be important for future experiments to determine whether supraphysiologic leptin levels can cause marked and sustained suppression of appetite and dysfunctional weight loss in a subpopulation of humans. If this occurred, it might be predicted to be in those individuals who easily resist obesity, consistent with these individuals having a robust lipostatic function of the hormone, as initially hypothesized (90) (Fig. 2). A narrow dose-response and quick saturation for leptin action may have resulted from evolutionary forces, as strong resistance to energy storage when food is abundant might be expected to have reduced fitness during periods of food insufficiency (85). Whether this evolutionary explanation is true or not, identification of mechanisms by which leptin physiologic action becomes saturated at relatively low leptin levels might suggest therapeutic approaches to enhancing leptin action to treat obesity. Two categories of potential mechanisms may be considered. In one, leptin action pathways inherently saturate over a narrow hormone range, beyond which further response is impossible. This could result from fully saturated receptor occupancy, or to rate-limiting steps downstream in signaling or effector pathways. As stated above, most hormones are associated with disease states of both deficiency and excess action. Having a dose-response curve that precludes supraphysiologic hormone levels from causing “excessive” hormone action is very uncommon in endocrine systems.

A second mechanism for leptin resistance could involve induction, as leptin levels rise, of one or more active inhibitors of leptin action within target cells, or further downstream in neuronal pathways. One such inhibitor that has been identified is suppressor of cytokine signaling suppressor of cytokine signaling (SOCS)3 (94, 95). Biochemical and genetic evidence in mice suggests this leptin-induced inhibitor of leptin signaling limits the ability of high leptin levels to prevent obesity (96). Parenthetically, SOCS proteins have also been shown to inhibit insulin signaling (97). Because key leptin signaling events take place in restricted cell types in the hypothalamus, it has been difficult to resolve the role of this or other molecules in clinical “leptin resistance.” Pharmacologic SOCS3 inhibitors capable of enhancing leptin action have not been developed, and genetic evidence that SOCS3 or other molecules cause leptin resistance in human obesity has also not been reported.

Two final points about leptin physiology deserve comment. Unlike insulin, where physiologic and biochemical dose-response curves have been extensively characterized *in vitro* and *in vivo* in animals and humans, *in vivo* physiologic dose responses to leptin in nonobese and obese animals and humans are very limited. There may be several reasons for this. The first is technical. Many insulin actions are quantitatively reflected in easily measured blood metabolites, and major target tissues are accessible for sampling to assess signaling. In contrast, leptin actions are complex and slower to develop, less readily quantified, and critical central nervous system sites of action are hard to sample, especially in humans. A second reason reflects the approach to early clinical development efforts. Unlike insulin, an approved drug that has been widely available for study, the company responsible for leptin’s early development made limited amounts available for investigator-initiated studies, perhaps out of concern that an adverse event could delay its commercial development path. Once the pivotal trial for obesity was declared a failure, the availability of leptin, and both its funding for and interest in its study, rapidly diminished.

Despite this disappointing outcome, there are reasons for optimism. As stated above, it may be possible to identify leptin-responsive subsets within the obese population, or to develop new approaches to enhancing the efficacy of the molecule, including coadministration with other hormones (98). Despite the evolutionary perspective that leptin’s physiology evolved to limit its ability to act as a potent antiobesity hormone, studies in rodents and humans have provided evidence that forced overfeeding can indeed engage physiologic pathways that resist obesity (99). One very recent study suggests that the marked suppression of appetite that follows forced overfeeding of mice cannot be due to leptin (100). Another study suggests that an as yet unidentified “gravitostat” signal independent of leptin regulates energy balance by sensing body weight (101). If one or more such hormones existed, understanding their role in physiology and disease might offer approaches to obesity treatment that leptin has so far not provided.

## Conclusion

Insulin and leptin, discovered 97 and 24 years ago, respectively, are two central hormones of metabolic physiology. They play key roles in signaling and orchestrating the transition between the fed and the starved or underfed states, although their respective physiologic roles are distinct. Their discoveries fundamentally changed our understanding of diabetes and obesity, two prevalent diseases.

Insulin may have been the more surprising of the two discoveries, having occurred at a time when science and technology were barely able to convincingly support the conclusion that a novel “internal secretion” had been discovered. It took a generation or more from “discovery” to elucidation of its structure and the first measurement of its levels in blood. Nevertheless, insulins discovery produced, with remarkable speed, a dramatically effective therapy for an important and increasingly prevalent disease. More broadly, insulin research has been a catalyst for the field of endocrinology. This includes the first characterization of a structurally defined peptide hormone, development of novel approaches to permit its measurement, path-breaking research to clarify its action through a plasma membrane receptor and downstream pathways, clinical development as a therapeutic with continuously enhanced formulation and purity, and, finally, its development as a recombinant hormone with diverse therapeutic analogs.

The two major forms of diabetes, types 1 and 2, are now known to be etiologically distinct disorders. In type 1, the *β* cells that produce insulin are destroyed by autoimmune attack. In type 2, the disease results from tissue resistance to insulin action combined with inadequate compensatory hypersecretion. For both type 1 and type 2 diabetes, there has been enormous growth of knowledge about the responsible pathophysiologic mechanisms, but this new knowledge has yet to produce precise definition of the molecular etiologies or treatments capable of reversing the underlying mechanisms.

The discovery of leptin took a completely different path, beginning with the study of a monogenic model of severe mouse obesity. Once effective genetic techniques and strategies were developed, eventual identification of the *ob* gene was never in doubt, although success at the outset was hardly assured. This discovery required great commitment, time, and skill in a rapidly evolving discipline, as well as teamwork. Once gene identification was accomplished, the demonstration that obesity was reversed by replacing the missing protein in animals (and humans) was a thrilling confirmation of Coleman’s hypothesis.

However, unlike the enormous clinical impact of insulin, the impact of leptin as a therapeutic has thus far been quite limited. Although leptin works brilliantly in those few who lack it, it has thus far failed in the general obese population in which hopes for its therapeutic efficacy were initially very high. Future research may expand the therapeutic role for leptin. Whether that happens or not, research on the neural circuits responsive to leptin have opened the door to critically important insights into the brain regulation of energy balance, now advancing rapidly through new techniques for precisely mapping and manipulating brain circuitry. As of now, the discovery of leptin has taught us more about a powerful peripheral signal that communicates with central neural energy balance circuits to signal the switch between starvation and the fed state than about mechanisms to control body weight or treat obesity.

The discoveries of both insulin and leptin, although arising within radically different scientific environments, reveal the importance of individual scientists driven by curiosity and ambition who are willing to pursue complex projects against great odds. These discoveries also reveal both the critical role of collaboration and the challenges to successfully sustaining collaborations, even when the goals of the work are fully accomplished. At a time when research is increasingly collaborative and interdisciplinary, we might now reconsider how the scientific community acknowledges scientific effort and awards credit for discovery under these evolving circumstances (102). Whatever the results of such a review, it is clear that these two discoveries have yielded enormous benefits both to science and to the fortunate patients whose lives have been transformed by novel and highly effective hormonal therapies. These discoveries will undoubtedly continue to be celebrated, as they should. As for the question of scientific credit for these discoveries, I would go back to the quote from Bliss as relates to insulin. There should—in the case of both insulin and leptin—be “glory enough for all” (1).

## Abbreviations

HHMI: Howard Hughes Medical Institute
SOCS: suppressor of cytokine signaling

## References

[1] Bliss M The Discovery of Insulin. Chicago, IL: University of Chicago Press; 1982.

[2] Farooqi IS, O’Rahilly S 20 Years of leptin: human disorders of leptin action. J Endocrinol. 2014;223(1):T63–T70. 25232148 10.1530/JOE-14-0480

[3] Bayliss WM, Starling EH The mechanism of pancreatic secretion. J Physiol. 1902;28(5):325–353. 16992627 10.1113/jphysiol.1902.sp000920 PMC1540572

[4] Banting FG, Best CH Pancreatic extracts. 1922. J Lab Clin Med. 1990;115(2):254–272. 2405086

[5] Banting FG, Best CH, MacLeod JJ The internal secretion of the pancreas. Am J Physiol. 1922;59:479.

[6] Li A J. B. Collip, A. M. Hanson and the isolation of the parathyroid hormone, or endocrines and enterprise. J Hist Med Allied Sci. 1992;47(4):405–438. 1469272 10.1093/jhmas/47.4.405

[7] Sanger F Chemistry of insulin; determination of the structure of insulin opens the way to greater understanding of life processes. Science. 1959;129(3359):1340–1344. 13658959 10.1126/science.129.3359.1340

[8] Yalow RS, Berson SA Immunoassay of plasma insulin in man. Diabetes. 1961;10(5):339–344. 13787083 10.2337/diab.10.5.339

[9] Freychet P, Roth J, Neville DM Jr Insulin receptors in the liver: specific binding of [^125^I]insulin to the plasma membrane and its relation to insulin bioactivity. Proc Natl Acad Sci USA. 1971;68(8):1833–1837. 4331561 10.1073/pnas.68.8.1833 PMC389303

[10] Flier JS, Kahn CR, Roth J, Bar RS Antibodies that impair insulin receptor binding in an unusual diabetic syndrome with severe insulin resistance. Science. 1975;190(4209):63–65. 170678 10.1126/science.170678

[11] Kasuga M, Karlsson FA, Kahn CR Insulin stimulates the phosphorylation of the 95,000-dalton subunit of its own receptor. Science. 1982;215(4529):185–187. 7031900 10.1126/science.7031900

[12] Ullrich A, Bell JR, Chen EY, Herrera R, Petruzzelli LM, Dull TJ, Gray A, Coussens L, Liao YC, Tsubokawa M, Mason A, Seeburg PH, Grunfeld C, Rosen OM, Ramachandran J Human insulin receptor and its relationship to the tyrosine kinase family of oncogenes. Nature. 1985;313(6005):756–761. 2983222 10.1038/313756a0

[13] Bray GA History of obesity. In: Williams G, Frühbeck G, eds. *Obesity: Science to Practice*. Chichester, UK: Wiley-Blackwell; 2009:3–18.

[14] Baskin ML, Ard J, Franklin F, Allison DB Prevalence of obesity in the United States. Obes Rev. 2005;6(1):5–7. 15655032 10.1111/j.1467-789X.2005.00165.x

[15] Afshin A, Forouzanfar MH, Reitsma MB, Sur P, Estep K, Lee A, Marczak L, Mokdad AH, Moradi-Lakeh M, Naghavi M, Salama JS, Vos T, Abate KH, Abbafati C, Ahmed MB, Al-Aly Z, Alkerwi A, Al-Raddadi R, Amare AT, Amberbir A, Amegah AK, Amini E, Amrock SM, Anjana RM, Ärnlöv J, Asayesh H, Banerjee A, Barac A, Baye E, Bennett DA, Beyene AS, Biadgilign S, Biryukov S, Bjertness E, Boneya DJ, Campos-Nonato I, Carrero JJ, Cecilio P, Cercy K, Ciobanu LG, Cornaby L, Damtew SA, Dandona L, Dandona R, Dharmaratne SD, Duncan BB, Eshrati B, Esteghamati A, Feigin VL, Fernandes JC, Fürst T, Gebrehiwot TT, Gold A, Gona PN, Goto A, Habtewold TD, Hadush KT, Hafezi-Nejad N, Hay SI, Horino M, Islami F, Kamal R, Kasaeian A, Katikireddi SV, Kengne AP, Kesavachandran CN, Khader YS, Khang YH, Khubchandani J, Kim D, Kim YJ, Kinfu Y, Kosen S, Ku T, Defo BK, Kumar GA, Larson HJ, Leinsalu M, Liang X, Lim SS, Liu P, Lopez AD, Lozano R, Majeed A, Malekzadeh R, Malta DC, Mazidi M, McAlinden C, McGarvey ST, Mengistu DT, Mensah GA, Mensink GBM, Mezgebe HB, Mirrakhimov EM, Mueller UO, Noubiap JJ, Obermeyer CM, Ogbo FA, Owolabi MO, Patton GC, Pourmalek F, Qorbani M, Rafay A, Rai RK, Ranabhat CL, Reinig N, Safiri S, Salomon JA, Sanabria JR, Santos IS, Sartorius B, Sawhney M, Schmidhuber J, Schutte AE, Schmidt MI, Sepanlou SG, Shamsizadeh M, Sheikhbahaei S, Shin MJ, Shiri R, Shiue I, Roba HS, Silva DAS, Silverberg JI, Singh JA, Stranges S, Swaminathan S, Tabarés-Seisdedos R, Tadese F, Tedla BA, Tegegne BS, Terkawi AS, Thakur JS, Tonelli M, Topor-Madry R, Tyrovolas S, Ukwaja KN, Uthman OA, Vaezghasemi M, Vasankari T, Vlassov VV, Vollset SE, Weiderpass E, Werdecker A, Wesana J, Westerman R, Yano Y, Yonemoto N, Yonga G, Zaidi Z, Zenebe ZM, Zipkin B, Murray CJ; GBD 2015 Obesity Collaborators Health effects of overweight and obesity in 195 countries over 25 years. N Engl J Med. 2017;377(1):13–27. 28604169 10.1056/NEJMoa1614362 PMC5477817

[16] Stunkard AJ, Foch TT, Hrubec Z A twin study of human obesity. JAMA. 1986;256(1):51–54. 3712713

[17] Ingalls AM, Dickie MM, Snell GD Obese, a new mutation in the house mouse. J Hered. 1950;41(12):317–318. 14824537 10.1093/oxfordjournals.jhered.a106073

[18] Robinson SW, Dinulescu DM, Cone RD Genetic models of obesity and energy balance in the mouse. Annu Rev Genet. 2000;34(1):687–745. 11092843 10.1146/annurev.genet.34.1.687

[19] Zhang Y, Proenca R, Maffei M, Barone M, Leopold L, Friedman JM Positional cloning of the mouse obese gene and its human homologue. Nature. 1994;372(6505):425–432. 7984236 10.1038/372425a0

[20] Lodish HF Molecular Cell Biology. 7th ed. New York, NY: W. H. Freeman and Co.; 2013.

[21] Coleman DL Obese and diabetes: two mutant genes causing diabetes-obesity syndromes in mice. Diabetologia. 1978;14(3):141–148. 350680 10.1007/BF00429772

[22] Coleman DL Effects of parabiosis of obese with diabetes and normal mice. Diabetologia. 1973;9(4):294–298. 4767369 10.1007/BF01221857

[23] Botstein D, White RL, Skolnick M, Davis RW Construction of a genetic linkage map in man using restriction fragment length polymorphisms. Am J Hum Genet. 1980;32(3):314–331. 6247908 PMC1686077

[24] Lander ES, Botstein D Mapping complex genetic traits in humans: new methods using a complete RFLP linkage map. Cold Spring Harb Symp Quant Biol. 1986;51(Pt 1):49–62. 2884068 10.1101/sqb.1986.051.01.007

[25] Monaco AP, Neve RL, Colletti-Feener C, Bertelson CJ, Kurnit DM, Kunkel LM Isolation of candidate cDNAs for portions of the Duchenne muscular dystrophy gene. Nature. 1986;323(6089):646–650. 3773991 10.1038/323646a0

[26] Collins FS, Riordan JR, Tsui LC The cystic fibrosis gene: isolation and significance. Hosp Pract (Off Ed). 1990;25(10):47–57. 10.1080/21548331.1990.11704019 1698801

[27] Friedman JM, Chung EY, Darnell JE Jr Gene expression during liver regeneration. J Mol Biol. 1984;179(1):37–53. 6502711 10.1016/0022-2836(84)90305-x

[28] Friedman JM, Babiss LE, Weiss M, Darnell JE Jr Hepatoma variants (C2) are defective for transcriptional and post-transcriptional actions from both endogenous and viral genomes. EMBO J. 1987;6(6):1727–1731. 2956094 10.1002/j.1460-2075.1987.tb02424.x PMC553548

[29] Shell ER *The Hungry Gene: The Science of Fat and the Future of Thin* New York, NY: Atlantic Monthly Press; 2002.

[30] Leibel RL, Hirsch J, Berry EM, Gruen RK Alterations in adipocyte free fatty acid re-esterification associated with obesity and weight reduction in man. Am J Clin Nutr. 1985;42(2):198–206. 4025192 10.1093/ajcn/42.2.198

[31] Leibel RL, Hirsch J Diminished energy requirements in reduced-obese patients. Metabolism. 1984;33(2):164–170. 6694559 10.1016/0026-0495(84)90130-6

[32] Edens NK, Leibel RL, Hirsch J Mechanism of free fatty acid re-esterification in human adipocytes in vitro. J Lipid Res. 1990;31(8):1423–1431. 2280183

[33] Bahary N, Zorich G, Pachter JE, Leibel RL, Friedman JM Molecular genetic linkage maps of mouse chromosomes 4 and 6. Genomics. 1991;11(1):33–47. 1684952 10.1016/0888-7543(91)90099-z

[34] Bahary N, Leibel RL, Joseph L, Friedman JM Molecular mapping of the mouse db mutation. Proc Natl Acad Sci USA. 1990;87(21):8642–8646. 1978328 10.1073/pnas.87.21.8642 PMC55013

[35] Bahary N, Pachter JE, Felman R, Leibel RL, Albright K, Cram S, Friedman JM Molecular mapping of mouse chromosomes 4 and 6: use of a flow-sorted Robertsonian chromosome. Genomics. 1992;13(3):761–769. 1639403 10.1016/0888-7543(92)90151-h

[36] Bahary N, Siegel DA, Walsh J, Zhang Y, Leopold L, Leibel R, Proenca R, Friedman JM Microdissection of proximal mouse chromosome 6: identification of RFLPs tightly linked to the *ob* mutation. Mamm Genome. 1993;4(9):511–515. 7906968 10.1007/BF00364786

[37] Halaas JL, Gajiwala KS, Maffei M, Cohen SL, Chait BT, Rabinowitz D, Lallone RL, Burley SK, Friedman JM Weight-reducing effects of the plasma protein encoded by the obese gene. Science. 1995;269(5223):543–546. 7624777 10.1126/science.7624777

[38] Pool R Fat: Fighting the Obesity Epidemic. New York, NY: Oxford University Press; 2001.

[39] Kolata GB Rethinking Thin: The New Science of Weight Loss–and the Myths and Realities of Dieting. 1st ed. New York, NY: Farrar, Straus, and Giroux; 2007.

[40] Merton RK The Matthew effect in science. The reward and communication systems of science are considered. Science. 1968;159(3810):56–63. 5634379

[41] Flier JS, Maratos-Flier E Lasker lauds leptin. Cell Metab. 2010;12(4):317–320. 20889124 10.1016/j.cmet.2010.09.008

[42] Friedman JM, Zhang Y, Proenca R, inventors; Rockefeller University, assignee. Modulators of body weight, corresponding nucleic acids and proteins, and diagnostic and therapeutic uses thereof. United States patent application 08/483,211, Publication No. US6309853. 30 October 2001.

[43] Kolata G Researchers find hormone causes a loss of weight: drug bonanza expected. *New York Times*. 26 July 1995:1.

[44] Farooqi IS, Jebb SA, Langmack G, Lawrence E, Cheetham CH, Prentice AM, Hughes IA, McCamish MA, O’Rahilly S Effects of recombinant leptin therapy in a child with congenital leptin deficiency. N Engl J Med. 1999;341(12):879–884. 10486419 10.1056/NEJM199909163411204

[45] Feudtner JC Bittersweet: Diabetes, Insulin, and the Transformation of Illness. Chapel Hill, NC: University of North Carolina Press; 2003.

[46] Nathan DM; DCCT/EDIC Research Group The diabetes control and complications trial/epidemiology of diabetes interventions and complications study at 30 years: overview. Diabetes Care. 2014;37(1):9–16. 24356592 10.2337/dc13-2112 PMC3867999

[47] Eisenbarth GS Type I diabetes mellitus. A chronic autoimmune disease. N Engl J Med. 1986;314(21):1360–1368. 3517648 10.1056/NEJM198605223142106

[48] Maahs DM, West NA, Lawrence JM, Mayer-Davis EJ Epidemiology of type 1 diabetes. Endocrinol Metab Clin North Am. 2010;39(3):481–497. 20723815 10.1016/j.ecl.2010.05.011 PMC2925303

[49] Keen H, Glynne A, Pickup JC, Viberti GC, Bilous RW, Jarrett RJ, Marsden R Human insulin produced by recombinant DNA technology: safety and hypoglycaemic potency in healthy men. Lancet. 1980;2(8191):398–401. 6105520 10.1016/s0140-6736(80)90443-2

[50] Mathieu C, Gillard P, Benhalima K Insulin analogues in type 1 diabetes mellitus: getting better all the time. Nat Rev Endocrinol. 2017;13(7):385–399. 28429780 10.1038/nrendo.2017.39

[51] Thabit H, Hovorka R Coming of age: the artificial pancreas for type 1 diabetes. Diabetologia. 2016;59(9):1795–1805. 27364997 10.1007/s00125-016-4022-4 PMC4969330

[52] Chang CA, Lawrence MC, Naziruddin B Current issues in allogeneic islet transplantation. Curr Opin Organ Transplant. 2017;22(5):437–443. 28692442 10.1097/MOT.0000000000000448

[53] Selvin E, Parrinello CM, Daya N, Bergenstal RM Trends in insulin use and diabetes control in the U.S.: 1988–1994 and 1999–2012. Diabetes Care. 2016;39(3):e33–e35. 26721815 10.2337/dc15-2229 PMC4764038

[54] Frederich RC, Hamann A, Anderson S, Löllmann B, Lowell BB, Flier JS Leptin levels reflect body lipid content in mice: evidence for diet-induced resistance to leptin action. Nat Med. 1995;1(12):1311–1314. 7489415 10.1038/nm1295-1311

[55] Considine RV, Sinha MK, Heiman ML, Kriauciunas A, Stephens TW, Nyce MR, Ohannesian JP, Marco CC, McKee LJ, Bauer TL, Caro JF Serum immunoreactive-leptin concentrations in normal-weight and obese humans. N Engl J Med. 1996;334(5):292–295. 8532024 10.1056/NEJM199602013340503

[56] Maffei M, Halaas J, Ravussin E, Pratley RE, Lee GH, Zhang Y, Fei H, Kim S, Lallone R, Ranganathan S, Kern PA, Friedman JM Leptin levels in human and rodent: measurement of plasma leptin and *ob* RNA in obese and weight-reduced subjects. Nat Med. 1995;1(11):1155–1161. 7584987 10.1038/nm1195-1155

[57] Reaven GM Role of insulin resistance in human disease (syndrome X): an expanded definition. Annu Rev Med. 1993;44(1):121–131. 8476236 10.1146/annurev.me.44.020193.001005

[58] Van Heek M, Compton DS, France CF, Tedesco RP, Fawzi AB, Graziano MP, Sybertz EJ, Strader CD, Davis HR Jr Diet-induced obese mice develop peripheral, but not central, resistance to leptin. J Clin Invest. 1997;99(3):385–390. 9022070 10.1172/JCI119171 PMC507810

[59] Heymsfield SB, Greenberg AS, Fujioka K, Dixon RM, Kushner R, Hunt T, Lubina JA, Patane J, Self B, Hunt P, McCamish M Recombinant leptin for weight loss in obese and lean adults: a randomized, controlled, dose-escalation trial. JAMA. 1999;282(16):1568–1575. 10546697 10.1001/jama.282.16.1568

[60] Ravussin E, Pratley RE, Maffei M, Wang H, Friedman JM, Bennett PH, Bogardus C Relatively low plasma leptin concentrations precede weight gain in Pima Indians. Nat Med. 1997;3(2):238–240. 9018247 10.1038/nm0297-238

[61] DePaoli A, Long A, Fine GM, Stewart M, O’Rahilly S Efficacy of metreleptin for weight loss in overweight and obese adults with low leptin levels. Diabetes. 2018;67(Suppl 1):LB77.

[62] Oral EA, Simha V, Ruiz E, Andewelt A, Premkumar A, Snell P, Wagner AJ, DePaoli AM, Reitman ML, Taylor SI, Gorden P, Garg A Leptin-replacement therapy for lipodystrophy. N Engl J Med. 2002;346(8):570–578. 11856796 10.1056/NEJMoa012437

[63] Brown RJ, Valencia A, Startzell M, Cochran E, Walter PJ, Garraffo HM, Cai H, Gharib AM, Ouwerkerk R, Courville AB Metreleptin improves insulin sensitivity independent of food intake in humans with lipodystrophy. J Clin Invest. 2018;128(8):3504–3516. 29723161 10.1172/JCI95476 PMC6063484

[64] Mittendorfer B, Horowitz JF, DePaoli AM, McCamish MA, Patterson BW, Klein S. Recombinant human leptin treatment does not improve insulin action in obese subjects with type 2 diabetes. *Diabetes*. 2011;**60**(5):1474–1477. 10.2337/db10-1302 PMC3292320 21411512

[65] Fujikawa T, Chuang JC, Sakata I, Ramadori G, Coppari R Leptin therapy improves insulin-deficient type 1 diabetes by CNS-dependent mechanisms in mice. *Proc Natl Acad Sci USA*. 2010;**107**(40):17391–17396. 10.1073/pnas.1008025107 PMC2951430 20855609

[66] Wang MY, Chen L, Clark GO, Lee Y, Stevens RD, Ilkayeva OR, Wenner BR, Bain JR, Charron MJ, Newgard CB Leptin therapy in insulin-deficient type I diabetes. *Proc Natl Acad Sci USA*. 2010;**107**(11):4813–4819. 10.1073/pnas.0909422107 PMC2841945 20194735

[67] Perry RJ, Zhang XM, Zhang D, Kumashiro N, Camporez JP, Cline GW, Rothman DL, Shulman GI Leptin reverses diabetes by suppression of the hypothalamic-pituitary-adrenal axis. Nat Med. 2014;20(7):759–763. 24929951 10.1038/nm.3579 PMC4344321

[68] Vasandani C, Clark GO, Adams-Huet B, Quittner C, Garg A Efficacy and safety of metreleptin therapy in patients with type 1 diabetes: a pilot study. Diabetes Care. 2017;40(5):694–697. 28223297 10.2337/dc16-1553

[69] Rosenbaum M, Goldsmith R, Bloomfield D, Magnano A, Weimer L, Heymsfield S, Gallagher D, Mayer L, Murphy E, Leibel RL Low-dose leptin reverses skeletal muscle, autonomic, and neuroendocrine adaptations to maintenance of reduced weight. J Clin Invest. 2005;115(12):3579–3586. 16322796 10.1172/JCI25977 PMC1297250

[70] Knuth ND, Johannsen DL, Tamboli RA, Marks-Shulman PA, Huizenga R, Chen KY, Abumrad NN, Ravussin E, Hall KD Metabolic adaptation following massive weight loss is related to the degree of energy imbalance and changes in circulating leptin. Obesity (Silver Spring). 2014;22(12):2563–2569. 25236175 10.1002/oby.20900 PMC4236233

[71] Tokarz VL, MacDonald PE, Klip A The cell biology of systemic insulin function. J Cell Biol. 2018;217(7):2273–2289. 29622564 10.1083/jcb.201802095 PMC6028526

[72] Meier JJ Insulin secretion. In: Jameson JL, De Groot LJ, eds. *Endocrinology: Adult and Pediatric*. 7th ed. Philadelphia, PA: Elsevier Saunders; 2016:546–555.e5.

[73] Boucher J, Kleinridders A, Kahn CR Insulin receptor signaling in normal and insulin-resistant states. Cold Spring Harb Perspect Biol. 2014;6(1):a009191. 24384568 10.1101/cshperspect.a009191 PMC3941218

[74] Martin BC, Warram JH, Krolewski AS, Bergman RN, Soeldner JS, Kahn CR Role of glucose and insulin resistance in development of type 2 diabetes mellitus: results of a 25-year follow-up study. Lancet. 1992;340(8825):925–929. 1357346 10.1016/0140-6736(92)92814-v

[75] Fuchsberger C, Flannick J, Teslovich TM, Mahajan A, Agarwala V, Gaulton KJ, Ma C, Fontanillas P, Moutsianas L, McCarthy DJ, Rivas MA, Perry JR, Sim X, Blackwell TW, Robertson NR, Rayner NW, Cingolani P, Locke AE, Tajes JF, Highland HM, Dupuis J, Chines PS, Lindgren CM, Hartl C, Jackson AU, Chen H, Huyghe JR, van de Bunt M, Pearson RD, Kumar A, Müller-Nurasyid M, Grarup N, Stringham HM, Gamazon ER, Lee J, Chen Y, Scott RA, Below JE, Chen P, Huang J, Go MJ, Stitzel ML, Pasko D, Parker SC, Varga TV, Green T, Beer NL, Day-Williams AG, Ferreira T, Fingerlin T, Horikoshi M, Hu C, Huh I, Ikram MK, Kim BJ, Kim Y, Kim YJ, Kwon MS, Lee J, Lee S, Lin KH, Maxwell TJ, Nagai Y, Wang X, Welch RP, Yoon J, Zhang W, Barzilai N, Voight BF, Han BG, Jenkinson CP, Kuulasmaa T, Kuusisto J, Manning A, Ng MCY, Palmer ND, Balkau B, Stančáková A, Abboud HE, Boeing H, Giedraitis V, Prabhakaran D, Gottesman O, Scott J, Carey J, Kwan P, Grant G, Smith JD, Neale BM, Purcell S, Butterworth AS, Howson JM, Lee HM, Lu Y, Kwak SH, Zhao W, Danesh J, Lam VKL, Park KS, Saleheen D, So WY, Tam CHT, Afzal U, Aguilar D, Arya R, Aung T, Chan E, Navarro C, Cheng CY, Palli D, Correa A, Curran JE, Rybin D, Farook VS, Fowler SP, Freedman BI, Griswold M, Hale DE, Hicks PJ, Khor CC, Kumar S, Lehne B, Thuillier D, Lim WY, Liu J, van der Schouw YT, Loh M, Musani SK, Puppala S, Scott WR, Yengo L, Tan ST, Taylor HA Jr, Thameem F, Wilson G Sr, Wong TY, Njølstad PR, Levy JC, Mangino M, Bonnycastle LL, Schwarzmayr T, Fadista J, Surdulescu GL, Herder C, Groves CJ, Wieland T, Bork-Jensen J, Brandslund I, Christensen C, Koistinen HA, Doney AS, Kinnunen L, Esko T, Farmer AJ, Hakaste L, Hodgkiss D, Kravic J, Lyssenko V, Hollensted M, Jørgensen ME, Jørgensen T, Ladenvall C, Justesen JM, Käräjämäki A, Kriebel J, Rathmann W, Lannfelt L, Lauritzen T, Narisu N, Linneberg A, Melander O, Milani L, Neville M, Orho-Melander M, Qi L, Qi Q, Roden M, Rolandsson O, Swift A, Rosengren AH, Stirrups K, Wood AR, Mihailov E, Blancher C, Carneiro MO, Maguire J, Poplin R, Shakir K, Fennell T, DePristo M, de Angelis MH, Deloukas P, Gjesing AP, Jun G, Nilsson P, Murphy J, Onofrio R, Thorand B, Hansen T, Meisinger C, Hu FB, Isomaa B, Karpe F, Liang L, Peters A, Huth C, O’Rahilly SP, Palmer CNA, Pedersen O, Rauramaa R, Tuomilehto J, Salomaa V, Watanabe RM, Syvänen AC, Bergman RN, Bharadwaj D, Bottinger EP, Cho YS, Chandak GR, Chan JC, Chia KS, Daly MJ, Ebrahim SB, Langenberg C, Elliott P, Jablonski KA, Lehman DM, Jia W, Ma RC, Pollin TI, Sandhu M, Tandon N, Froguel P, Barroso I, Teo YY, Zeggini E, Loos RJF, Small KS, Ried JS, DeFronzo RA, Grallert H, Glaser B, Metspalu A, Wareham NJ, Walker M, Banks E, Gieger C, Ingelsson E, Im HK, Illig T, Franks PW, Buck G, Trakalo J, Buck D, Prokopenko I, Mägi R, Lind L, Farjoun Y, Owen KR, Gloyn AL, Strauch K, Tuomi T, Kooner JS, Lee JY, Park T, Donnelly P, Morris AD, Hattersley AT, Bowden DW, Collins FS, Atzmon G, Chambers JC, Spector TD, Laakso M, Strom TM, Bell GI, Blangero J, Duggirala R, Tai ES, McVean G, Hanis CL, Wilson JG, Seielstad M, Frayling TM, Meigs JB, Cox NJ, Sladek R, Lander ES, Gabriel S, Burtt NP, Mohlke KL, Meitinger T, Groop L, Abecasis G, Florez JC, Scott LJ, Morris AP, Kang HM, Boehnke M, Altshuler D, McCarthy MI The genetic architecture of type 2 diabetes. Nature. 2016;536(7614):41–47. 27398621 10.1038/nature18642 PMC5034897

[76] Moller DE, Yokota A, White MF, Pazianos AG, Flier JS A naturally occurring mutation of insulin receptor alanine 1134 impairs tyrosine kinase function and is associated with dominantly inherited insulin resistance. J Biol Chem. 1990;265(25):14979–14985. 2168397

[77] Melvin A, O’Rahilly S, Savage DB Genetic syndromes of severe insulin resistance. Curr Opin Genet Dev. 2018;50:60–67. 29477938 10.1016/j.gde.2018.02.002

[78] Czech MP Insulin action and resistance in obesity and type 2 diabetes. Nat Med. 2017;23(7):804–814. 28697184 10.1038/nm.4350 PMC6048953

[79] Pernicova I, Korbonits M Metformin—mode of action and clinical implications for diabetes and cancer. Nat Rev Endocrinol. 2014;10(3):143–156. 24393785 10.1038/nrendo.2013.256

[80] Slieker LJ, Sloop KW, Surface PL, Kriauciunas A, LaQuier F, Manetta J, Bue-Valleskey J, Stephens TW Regulation of expression of *ob* mRNA and protein by glucocorticoids and cAMP. J Biol Chem. 1996;271(10):5301–5304. 8621378 10.1074/jbc.271.10.5301

[81] Barr VA, Malide D, Zarnowski MJ, Taylor SI, Cushman SW Insulin stimulates both leptin secretion and production by rat white adipose tissue. Endocrinology. 1997;138(10):4463–4472. 9322964 10.1210/endo.138.10.5451

[82] Mantzoros CS, Qu D, Frederich RC, Susulic VS, Lowell BB, Maratos-Flier E, Flier JS Activation of β_3_ adrenergic receptors suppresses leptin expression and mediates a leptin-independent inhibition of food intake in mice. Diabetes. 1996;45(7):909–914. 8666142 10.2337/diab.45.7.909

[83] Caron A, Dungan Lemko HM, Castorena CM, Fujikawa T, Lee S, Lord CC, Ahmed N, Lee CE, Holland WL, Liu C, Elmquist JK POMC neurons expressing leptin receptors coordinate metabolic responses to fasting via suppression of leptin levels. eLife. 2018;7:e33710. 29528284 10.7554/eLife.33710 PMC5866097

[84] Ahima RS, Prabakaran D, Mantzoros C, Qu D, Lowell B, Maratos-Flier E, Flier JS Role of leptin in the neuroendocrine response to fasting. Nature. 1996;382(6588):250–252. 8717038 10.1038/382250a0

[85] Flier JS Clinical review 94. What’s in a name? In search of leptin’s physiologic role. J Clin Endocrinol Metab. 1998;83(5):1407–1413. 9589630 10.1210/jcem.83.5.4779

[86] Welt CK, Chan JL, Bullen J, Murphy R, Smith P, DePaoli AM, Karalis A, Mantzoros CS Recombinant human leptin in women with hypothalamic amenorrhea. N Engl J Med. 2004;351(10):987–997. 15342807 10.1056/NEJMoa040388

[87] Rosenbaum M, Murphy EM, Heymsfield SB, Matthews DE, Leibel RL Low dose leptin administration reverses effects of sustained weight-reduction on energy expenditure and circulating concentrations of thyroid hormones. J Clin Endocrinol Metab. 2002;87(5):2391–2394. 11994393 10.1210/jcem.87.5.8628

[88] Flak JN, Myers MG Jr Minireview: CNS mechanisms of leptin action. Mol Endocrinol. 2016;30(1):3–12. 26484582 10.1210/me.2015-1232 PMC4695630

[89] Loh K, Zhang L, Brandon A, Wang Q, Begg D, Qi Y, Fu M, Kulkarni R, Teo J, Baldock P, Brüning JC, Cooney G, Neely G, Herzog H Insulin controls food intake and energy balance via NPY neurons. Mol Metab. 2017;6(6):574–584. 28580287 10.1016/j.molmet.2017.03.013 PMC5444095

[90] Friedman JM, Halaas JL Leptin and the regulation of body weight in mammals. Nature. 1998;395(6704):763–770. 9796811 10.1038/27376

[91] Olefsky JM, Kolterman OG Mechanisms of insulin resistance in obesity and noninsulin-dependent (type II) diabetes. Am J Med. 1981;70(1):151–168. 7006388 10.1016/0002-9343(81)90422-8

[92] Halaas JL, Boozer C, Blair-West J, Fidahusein N, Denton DA, Friedman JM Physiological response to long-term peripheral and central leptin infusion in lean and obese mice. Proc Natl Acad Sci USA. 1997;94(16):8878–8883. 9238071 10.1073/pnas.94.16.8878 PMC23177

[93] Flier JS, Maratos-Flier E Leptin’s physiologic role: does the emperor of energy balance have no clothes? Cell Metab. 2017;26(1):24–26. 28648981 10.1016/j.cmet.2017.05.013

[94] Bjørbæk C, Elmquist JK, Frantz JD, Shoelson SE, Flier JS Identification of SOCS-3 as a potential mediator of central leptin resistance. Mol Cell. 1998;1(4):619–625. 9660946 10.1016/s1097-2765(00)80062-3

[95] Bjørbæk C, El-Haschimi K, Frantz JD, Flier JS The role of SOCS-3 in leptin signaling and leptin resistance. J Biol Chem. 1999;274(42):30059–30065. 10514492 10.1074/jbc.274.42.30059

[96] Howard JK, Cave BJ, Oksanen LJ, Tzameli I, Bjørbaek C, Flier JS Enhanced leptin sensitivity and attenuation of diet-induced obesity in mice with haploinsufficiency of Socs3. Nat Med. 2004;10(7):734–738. 15220914 10.1038/nm1072

[97] Emanuelli B, Peraldi P, Filloux C, Sawka-Verhelle D, Hilton D, Van Obberghen E SOCS-3 is an insulin-induced negative regulator of insulin signaling. J Biol Chem. 2000;275(21):15985–15991. 10821852 10.1074/jbc.275.21.15985

[98] Roth JD, Roland BL, Cole RL, Trevaskis JL, Weyer C, Koda JE, Anderson CM, Parkes DG, Baron AD Leptin responsiveness restored by amylin agonism in diet-induced obesity: evidence from nonclinical and clinical studies. Proc Natl Acad Sci USA. 2008;105(20):7257–7262. 18458326 10.1073/pnas.0706473105 PMC2438237

[99] Ravussin Y, Leibel RL, Ferrante AW Jr A missing link in body weight homeostasis: the catabolic signal of the overfed state. Cell Metab. 2014;20(4):565–572. 25295786 10.1016/j.cmet.2014.09.002 PMC4191848

[100] Ravussin Y, Edwin E, Gallop M, Xu L, Bartolomé A, Kraakman MJ, LeDuc CA, Ferrante AW Jr Evidence for a non-leptin system that defends against weight gain in overfeeding. Cell Metab. 2018;28(2):289–299.e5. 29937378 10.1016/j.cmet.2018.05.029 PMC6082718

[101] Jansson J-O, Palsdottir V, Hägg DA, Schéle E, Dickson SL, Anesten F, Bake T, Montelius M, Bellman J, Johansson ME, Cone RD, Drucker DJ, Wu J, Aleksic B, Törnqvist AE, Sjögren K, Gustafsson JÅ, Windahl SH, Ohlsson C Body weight homeostat that regulates fat mass independently of leptin in rats and mice. Proc Natl Acad Sci USA. 2018;115(2):427–432. 29279372 10.1073/pnas.1715687114 PMC5777058

[102] Borenstein J, Shamoo AE Rethinking authorship in the era of collaborative research. Account Res. 2015;22(5):267–283. 25928178 10.1080/08989621.2014.968277

